# Radish (*Raphanus sativus*) and Diabetes

**DOI:** 10.3390/nu9091014

**Published:** 2017-09-14

**Authors:** Saleem Ali Banihani

**Affiliations:** Department of Medical Laboratory Sciences, Jordan University of Science and Technology, Irbid 22110, Jordan; sabanihani@just.edu.jo; Tel.: +962-2720-1000

**Keywords:** radish, diabetes, antioxidants, *Raphanus sativus*, glucose hemostasis

## Abstract

For more than three decades, various in vitro and in vivo studies have linked radishes with diabetes, though this link has not been discussed. This review systematically addresses and summarizes the effect of radishes on diabetes. We searched the Web of Science, PubMed, and EMBASE databases for English language articles from June 1987 through May 2017 using the key words “radish” and “diabetes,” and the references from particular reports were also considered if relevant. In summary, radish has been identified as having antidiabetic effects, making it favorable for those with diabetic conditions. This may be due to its ability to enhance the antioxidant defense mechanism and reduce the accumulation of free radicals, affect hormonal-induced glucose hemostasis, promote glucose uptake and energy metabolism, and reduce glucose absorption in the intestine. However, this summary requires further confirmation in research in vivo studies and clinical trials.

## 1. Introduction

Radish (*Raphanus sativus*) is a root vegetable grown and consumed all over the world and is considered part of the human diet, even though it is not common among some populations. Usually, people eat radishes raw as a crunchy vegetable, mainly in salad, while it also appears in many European dishes. Some people, at least in the Middle East, prefer to drink its juice in pursuit of certain health benefits. Radishes have different skin colors (red, purple, black, yellow, and white through pink), while its flesh is typically white. In addition, the edible root of radish varies in its flavor, size, and length throughout the world. 

In Unani, Greeko-Arab, and Indian folk medicine, radish is used as a household remedy for the treatment of many diseases such as jaundice, gallstone, liver diseases, rectal prolapse, indigestion, and other gastric pains [1,2]. In general, radish contains carbohydrates, sugars, dietary fibers, protein, and even some fat and fluoride [3]. In addition, it contains various water-soluble vitamins (B_1_, B_2_, B_3_, B_5_, B_6_, B_9_, and C) and minerals (calcium, iron, magnesium, manganese zinc, potassium, and phosphorous) [3]. Above and beyond, radish was found to have unique bioactive compounds that have been recently recognized to have potential health benefits to humans. The main bioactive compounds that have been quantified in radish are glucosinolates (e.g., glucoraphanin, glucoraphanin, 4-hydroxyglucobrassicin, glucoerucin, glucoraphasatin, glucobrassicin, 4-methioxyglucobrassicin, and neoglucobrassicin) and isothiocyanates (e.g., sulforaphene, sulforaphane, and indole-3-carbinol) [4,5,6]. It is important to mention that these isothiocyanates are breakdown products resulting from the enzymatic hydrolysis of glucosinolates by the enzyme myrosinase, which is present in radish [4,7]. In fact, the myrosinase enzymatic activity is also present in the bacterial microflora of the human colon. 

Lately, several research studies have revealed the many health properties of radish, and some of these studies have even had a chance to describe or suggest, albeit partially, the molecular features of these effects. For example, methylisogermabullone (C_23_H_31_O_5_NS, MW 433), a bioactive compound isolated from radish, was found to stimulate the small bowel mobility by activating the acetylcholinergic receptors [2]. 4-(Methylthio)-3-butenyl isothiocyanate was found to induce apoptosis in human colon cancer cells, hence reducing the abnormal cell growth [8]. Moreover, the spicy flavor of radish is due to certain chemical substances (e.g., myrosinase, glucosinolate, and isothiocyanate) made by this plant [9], some of which were suggested to have anticancer activities. A cohort study (*n* = 11,405 male participants) in Germany (2009) revealed an adverse correlation between dietary intake of glucosinolate and the risk of prostate cancer [10]. 4-Methylsulfinyl-3-butenyl isothiocyanate present in radish was found to induce apoptosis in human lung cancer cells [11]. A very recent study showed that sulforaphene, an isothiocyanate found in radish, inhibits proliferation of breast cancer cells [12]. 

Furthermore, an in vitro study by Suh et al. (2006) demonstrated that white radish taproot extract inhibited the abnormal proliferation of vascular smooth muscle cells [4]. The study by Kim et al. (2014) showed that the Asian white radish of an edible solid taproot has anti-inflammatory and anti-cancerous activities [13]. Additionally, in 2014, Castro-Torres et al. revealed that black radish had the potential to diminish cholesterol level and to reduce cholesterol gallstones in mice [14]. Furthermore, crude aqueous extract of black radish (also named Spanish black radish) induced detoxification enzymes such as quinone reductase, cytochrome P450, thioredoxin reductase 1, and heme oxygenase 1 in the HepG2 human hepatoma cell line [15]. Results from the same study demonstrated that the bioactive metabolite 4-methylthio-3-butenyl isothiocyanate (raphasatin) is a potent inducer for this detoxification catalytic activity [15]. A recent in vivo system study showed that radish extract prevented cadmium-induced biochemical and immunotoxic alterations [16]. 

Diabetes is a global health quandary, as the number of diabetic people is increasing every year. It is a serious illness characterized by uncontrolled glucose hemostasis [17]. Currently, diabetes is one of the top leading causes of death in humans [18]. Therefore, studies and reports that look into effective oral therapies or household remedies to manage diabetes are still direly needed. The World Health Organization expert committee recommended exploring and considering antidiabetic agents of plant origins, given that the synthetic hypoglycemic drugs are, most of the time, a double-edged sword and may induce undesirable side effects such as gastrointestinal irritation, nausea, and thyrotropin suppression [19,20].

In particular, since the late 1980s, various basic as well as clinical research studies have established connections, directly and indirectly, between radish and diabetes. However, whether narratively or systematically, such connection has not yet been reviewed. This review systematically addresses and summarizes the effect of radish on diabetes. To achieve this contribution, we searched the Web of Science, PubMed, and EMBASE databases for English language articles from June 1987 through May 2017 using the key words “radish” and “diabetes.” In addition, the references from particular published articles were also reviewed, only if relevant.

## 2. The Effect of Radish on Diabetic Conditions

To date, almost all published research studies that have directly connected radish with diabetes are non-clinical (i.e., in vitro or in vivo system studies). Table 1 presents a summary of the main studies done on radish and its extracts and their reported effects on diabetic parameters. These presented effects confirm the presence of the hypoglycemic effect of radish. The water-soluble radish extract showed an advantage in the hypoglycemic response over the fat-soluble extract, while the fat-soluble extract was found to play a role in lipid metabolism [21]. It was suggested that the water-soluble extract contains insulin-like components (e.g., polyphenolic substances [22]) or glucosidase-inhibiting components [21].

Further, streptozotocin-induced diabetic rats fed with the Egyptian radish at 10% for 6 weeks had significant changes in the histopathological examination of the pancreas [24]. Furthermore, radish significantly reduced the starch induced-postprandial glycemic load, suggesting that it has a potent antidiabetic activity [25]. This evidence confirms that radishes and radish extracts are favorable to diabetes and diabetic conditions.

As indicated in Table 1, diabetic rats supplemented lyophilized radish root-juice at 300 mg kg^−1^ of body weight had lower blood glucose level by about 33.4%, after 6 h of fasting. In humans, this therapeutic dose is equal to ~48.39 mg kg^−1^ of body weight [26]. Accordingly, for example, the effective therapeutic dose for a man of 60 kg is approximately 2903 mg of lyophilized radish root-juice; this amount is extracted from ~0.726 kg of radish [1].

## 3. Mechanistic Studies

Several studies have revealed the chemical and molecular aspects of antidiabetic activity of radish and its different extracts; however, all of these studies have been non-clinical.

### 3.1. Glucose-Regulatory Hormones

Some studies have suggested that radish may exert its antidiabetic activity by affecting certain hormones that affect glucose hemostasis. For example, an in vitro study on 3T3-L1 adipocytes found that ethanol extract of radish enhances the production of adiponectin (a peptide hormone that modulates the regulation of glucose and fatty acids [27]) [28].

Japanese radish sprouts (2.5–5% of the diet) were found to reduce the level of plasma insulin in normal and streptozotocin-induced diabetic rats [23]. This reduction suggests that the hypoglycemic effect of radish sprouts were brought about by ameliorating insulin sensitivity or exerting insulin-like effect, but not by enhancing the production of insulin [23]. Antioxidants might be the chemical components that are responsible for such hypoglycemic response. For example, catechin, a phenolic compound present in radish, significantly enhanced insulin secretion [29].

### 3.2. Diabetes-Induced Oxidative Damage

A number of studies have suggested that the antidiabetic activity of radish may be due to its ability to enhance the antioxidant defense mechanism and reduce oxidative stress, which is an imbalance between reactive oxygen species and antioxidants in cellular systems [30,31,32,33]. The partially purified superoxide dismutase-like activity protein was found to increase the in vitro glucose uptake by the erythrocytes of diabetic patients [34]. As evidence, the results from this study demonstrated a significant reduction in the concentration of malondialdehyde (a biomarker of lipid peroxidation) and thus a reduction in the oxidative stress formed in erythrocytes [34]. Methanolic extract of radish root (~40–160 mg kg^−1^ of body weight) inhibited in vivo lipid peroxidation in albino rats, and in vitro cumene hydroperoxide induced lipid peroxidation [35]. It was found to strengthen the endogenous antioxidants such as glutathione and catalase [35]. Indirectly, squeezed juice from the black radish root has metal-chelating activity (i.e., copper-chelating activity), which, consequently, reduces reactive oxygen species generation, mainly by hindering Fenton’s reaction [36,37]. Alternatively, radish leaf juice has displayed potential antioxidant behavior against hydrogen peroxide-induced oxidative hemolysis in rat red blood cells [38].

In addition, radish was found to contain coenzyme Q10 (also named ubiquinone), a fat-soluble antioxidant coenzyme and a component of the electron transport chain in human mitochondria, which prevents the development of type 2 diabetes [39,40,41,42]. The in vivo system study published by Lee et al. (2014) suggested that coenzyme Q10 at ~1600–2000 mg kg^−1^ of body weight in mice weighing 25–30 g, which equals ~130.1–162.6 mg/kg of body weight in humans, could be a promising therapeutic approach for ameliorating oxidative stress in glaucomatous neurodegeneration [43]. Later study on streptozotocin-induced diabetic rats showed that both forms of coenzyme Q10 (ubiquinol-10 and ubiquinone-10) decreased the oxidative stress state, and the long-term administration of coenzyme Q10 (4 weeks) appeared to be safe [44].

Moreover, radish contains anthocyanins, potent antioxidant flavonoids that have been found to be favorable for improving diabetic conditions [45,46]. The anthocyanin subtype that is present in radish is pelargonidin [47]. It was found that oral administration of pelargonidin at 10 mg/kg of body weight (~1.62 mg/kg in adult humans [26]) prevents diabetic neuropathic hyperalgesia in streptozotocin-induced diabetic rats via the decrease in oxidative stress [48]. It was found that 100 g of common red radish (~11 radishes; 1–1.25″ diameter each) contains approximately 63.1 mg pelargonidin [47]. Accordingly, in diabetic conditions, a therapeutic dose of pelargonidin for a man of 60 kg can be achieved at 97.2 mg, which is equal to ~154 g radish (~16.94 radishes of ~1–1.25″ diameter each). A very recent study demonstrated that pelargonidin may improve the redox state of HepG2 cells via reducing reactive oxygen species generation, increasing antioxidant enzymes activities and decreasing thiobarbituric acid-reactive moiety formation [49]. A study conducted by Graf et al. (2013) found that anthocyanin-rich grape bilberry juice (1551 mg anthocyanins L^−1^) lowers resistin and leptin, adipokines involved in the development of metabolic diseases including type 2 diabetes, in Fischer rats [50].

Furthermore, isothiocyanates, particularly sulforaphane (~5 µM), were found to induce phase II antioxidant enzymes such as glutathione transferase, heme oxygenase, NAD(P)H: quinone reductase, epoxide hydrolase, and UDP-glucuronosyltransferase [51]. The induction of these enzymes is very beneficial in diabetic conditions as it reduces the accumulation of free radicals and hence oxidative damage [52]. 

Polyphenolic content in radish was estimated to be approximately in the range 13.18–63.54 mg g^−1^ dry weight [53]. In water extract, catechin was found to be the most abundant phenolic compound, while sinapic acid was found to be the predominant phenolic compound in methanolic and hexane extract. The methanolic extract exhibited moderate metal chelating activity, strong ferric reducing ability, and strong free-radical scavenging activity.

Above and beyond, due to its short development cycle, radish has been found to be a good choice as a selenium-enriched diet for humans [54]. Indeed, selenium-enriched radish enhanced glutathione peroxidase and glutathione S-transferase activities in the lungs and the livers of rats equally [55]. Such catalytic promotion may prevent the possible oxidative injury to cells, thus delaying the onset of reactive oxygen species-mediated aging diseases, including type 2 diabetes [54]. 

### 3.3. Glucose Uptake or Absorption and Energy Metabolism

In addition, studies have revealed that radish and its bioactive compounds may exert the antidiabetic activity via modulating glucose uptake. It has been suggested that oral administration of radish seeds (also called Raphani semen) improves insulin resistance in Sprague-Dawley rats, mainly by decreasing blood viscosity, which consequently increases the binding affinity between insulin receptors, hence enhancing glucose uptake [56]. 

Alternatively, studies have suggested a major role for tumor necrosis factor-alpha in insulin resistance [57,58]. It has been shown that tumor necrosis factor-alpha expression is increased in adipose tissues, and its neutralization is considered as one model to improve insulin sensitivity, which occurs by enhancing the activity of tyrosine-kinase insulin receptors [57,59]. In 2010, Okada and co-workers found that ethanol extract of radish seeds (2 µL significantly reduced the levels of tumor necrosis factor-alpha in 3T3-L1 adipocytes, suggesting a beneficial effect of radish in non-insulin dependent diabetes mellitus conditions [28].

Additionally, the study by Baenas and co-workers on *Drosophila mlanogaster* demonstrated that the consumption of radish sprouts (10.6 g L^−1^, for 10 days) may affect the energy metabolism by increasing the expression of *spargel* (the Drosophila homolog of the mammalian peroxisome proliferator-activated receptor γ-coactivator 1α) which is considered as a key player in mitochondrial biogenesis [4]. This process may postpone the development of aging diseases, including type 2 diabetes [4,60]. 

### 3.4. Radish Reduces Glucose Absorption

Furthermore, the aqueous extract of radish inhibited both α-amylase and α-glucosidase enzymes in vitro [4,61]. Aqueous extract of radish leaves at 10 mg mL^−1^ was found to significantly inhibit α-glucosidase activity [62]. It is well-known that these enzymes are required for the degradation of poly-and oligosaccharides in the intestine before absorption [4]. Therefore, such enzymatic inhibition may reduce the amount of glucose absorbed, which could be effective for the management and prevention of diabetes [4].

## 4. Summary and Future Perspectives 

**Figure 1:**
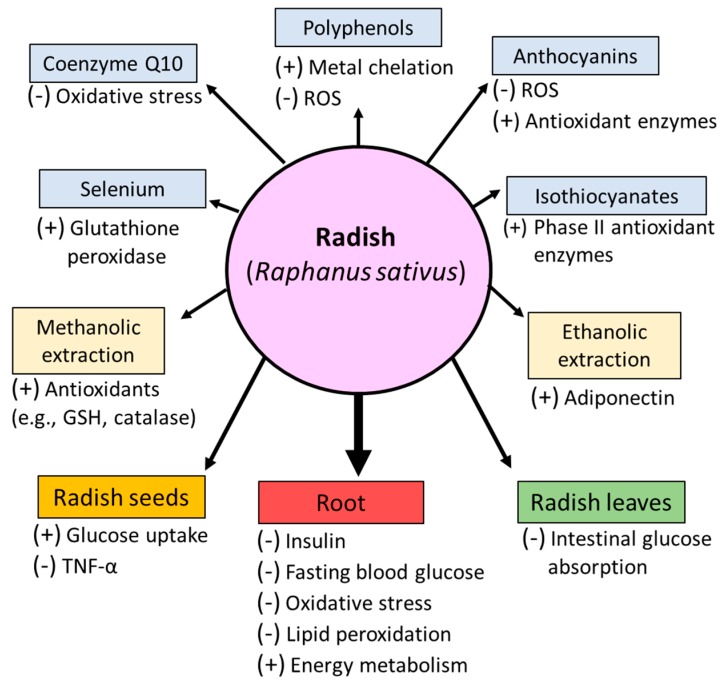
Effect of radish and some of its bioactive components on type 2 diabetic conditions. (+) increase; (−) decrease; ROS: reactive oxygen species.

In summary, radish root appears to have an antidiabetic effect and appears to be very beneficial in diabetic conditions (Figure 1). These antidiabetic properties may be due to its ability to enhance the antioxidant defense mechanism and decrease oxidative stress and lipid peroxidation, improve hormonal-induced glucose hemostasis, promote glucose uptake and energy metabolism, and reduce glucose absorption in the intestine.

The other anatomical parts of radish were found to be beneficial for diabetic conditions. Radish seeds were found to ameliorate insulin resistance and enhance glucose uptake, while radish leaves were found to reduce intestinal glucose absorption. This summary requires further confirmation through clinical studies.

## Figures and Tables

**Table 1:** A summary of the studies done on radish and its extracts and their reported effects on diabetic parameters.

Treatment	Dose	Duration	Population	Effect on Diabetic Parameters	Reference
Radish sprouts	2.5–5% of the diet	21 days	Streptozotocin-induced diabetic rats	(−) Glucose (−) Fructosamine (−) Insulin (−) Triglycerides	[23]
Water-soluble radish extract	2.2% of the diet	3 weeks	Streptozotocin-induced diabetic rats	(−) Glucose (−) Glycoalbumin (−) Fructosamine	[21]
Radish root juice	300 mg kg^−1^ of body weight	6 h 3 h-GTT	Normal rats	(−) Fasting blood glucose (−) Glucose–using glucose tolerance test (GTT)	[1]
Radish root juice	300 mg kg^−1^ of body weight	6 h 3 h-GTT	Sub- and mild diabetic rats	(−) Fasting blood glucose (−) Glucose–using glucose tolerance test	[1]
Egyptian radish	10% of the diet	6 weeks	Streptozotocin-induced diabetic rats	(−) Fasting blood glucose	[24]
Lyophilized radish sprouts	10.6 g/L	10 days	Drosophila melanogaster	(−) Glucose content (+) Spargel expression- (drosophila homolog of the mammalian PPARγ-coactivator 1 α).	[4]

(−) decrease; (+) increase.
